# Critical Role of TCF-1 in Repression of the IL-17 Gene

**DOI:** 10.1371/journal.pone.0024768

**Published:** 2011-09-14

**Authors:** Jian Ma, Ruiqing Wang, Xianfeng Fang, Yan Ding, Zuoming Sun

**Affiliations:** Division of Immunology, Beckman Research Institute of the City of Hope, Duarte, California, United States of America; University of Crete, Greece

## Abstract

Overwhelming activation of IL-17, a gene involved in inflammation, leads to exaggerated Th17 responses associated with numerous autoimmune conditions, such as experimental autoimmune encephalomyelitis (EAE). Here we show that TCF-1 is a critical factor to repress IL-17 gene locus by chromatin modifications during T cell development. Deletion of TCF-1 resulted in increased IL-17 gene expression both in thymus and peripheral T cells, which led to enhanced Th17 differentiation. As a result, *TCF-1^-/-^* mice were susceptible to Th17-dependent EAE induction. *Rag1^-/-^* mice reconstituted with *TCF-1^-/-^* T cells were also susceptible to EAE, indicating TCF-1 is intrinsically required to repress IL-17. However, expression of wild-type TCF-1 or dominant negative TCF-1 did not interfere with Th17 differentiation in mature T cells. Furthermore, expression of TCF-1 in *TCF-1^-/-^* T cells could not restore Th17 differentiation to wild-type levels, indicating that TCF-1 cannot affect IL-17 production at the mature T cell stage. This is also supported by the normal up-regulation or activation in mature *TCF-1^-/-^* T cells of factors known to regulate Th17 differentiation, including RORγt and Stat3. We observed hyperacetylation together with trimethylation of Lys-4 at the IL-17 locus in *TCF-1^-/-^* thymocytes, two epigenetic modifications indicating an open active state of the gene. Such epigenetic modifications were preserved even when *TCF-1^-/-^* T cells migrated out of thymus. Therefore, TCF-1 mediates an active process to repress IL-17 gene expression via epigenetic modifications during T cell development. This TCF-1-mediated repression of IL-17 is critical for peripheral T cells to generate balanced immune responses.

## Introduction

In response to antigen stimulation, naïve T cells have the potential to differentiate into multiple lineages of T helpers such as Th1, Th2, Th17 and regulatory T cells (Treg). Because the different T helpers perform distinct, sometimes even opposite, functions [1], balanced differentiation to different T helpers is critical for maintaining properly immune responses. Inappropriately enhanced or reduced potential for forming specific types of T helpers leads to either autoimmunity or immunodeficiency. Many studies have focused on how the cytokine environment regulates T cell differentiation into different types of T helpers; however, little is known about whether and how the developmental process programs T cells to generate balanced immune responses once they migrate out to periphery. Positive and negative selection during development of T cells in the thymus ensures that mature T cells are responsive only to foreign but not self-antigens [2]. Another function of T cell development in the thymus is to program T cells such that they have unbiased potential to form different lineages of T helpers, as biased potential to form a specific type of T helper leads to unbalanced immune responses.

Identification of the Th17 lineage has changed the traditional Th1/Th2 paradigm by adding a new lineage of T helpers [3]. In 2003, *Cua et al*. made the seminal observation that IL-23, but not the Th1 cytokine IL-12, was critical for the development of experimental autoimmune encephalomyelitis (EAE), which had long been believed to be a Th1-dependent autoimmune disease [4]. IL-23 was required for the expansion of IL-17-producing pathogenic T cells, which induced EAE when adoptively transferred to naïve mice [5]. Th17 cells produce IL-17, IL-21 and IL-22 cytokines [6], and IL-17 induces massive tissue inflammation due to broad distribution of its receptors on both immune and nonimmune cells [6]. In addition to EAE, Th17 has also been identified as the pathogenic T cell in an animal model of collagen-induced arthritis [7] and inflammatory bowel disease (IBD) [8]. Circumstantial evidence has also accumulated suggesting the pathologenic role of Th17 cells in multiple human autoimmune disorders, including multiple sclerosis [9], rheumatoid arthritis [10], asthma [11], IBD [12] and psoriasis [13]. A greater understanding of the mechanisms responsible for regulating the differentiation of Th17 will therefore facilitate the development of treatments that target Th17-mediated autoimmunity [14]. Stimulation of naïve T cells in the presence of TGFβ and IL-6 is sufficient to form IL-17-producing T cells [15], and IL-6 activates Stat3, which is essential for formation of Th17 cells [16]. In addition, IL-6 and TGFβ together are required to induce RORγt, a master regulator of Th17. T cells deficient in RORγt fail to become IL-17 producing cells, even in the presence of TGFβ and IL-6 [17]. So far, almost all the factors known to interfere with Th17 differentiation do so by direct or indirect regulation of RORγt expression [18]. Peripheral T cells in *RORγt^-/-^* mice can form Th17 cells once RORγt is provided [17], suggesting that RORγt is not required for the thymic development of T cells with the potential to form Th17 cells. However, it is still unclear whether the potential of Th17 formation is regulated during T cell development in thymus.

T cell factor 1 (TCF-1) is a transcription factor that is enriched in hematopoietic cells and plays an important role in T cell development in the thymus [19], [20], [21], [22], [23], [24] as well as T cell differentiation in the periphery [25]. TCF-1 plays an important role in the CD4^-^CD8^-^ double negative to CD4^+^CD8^+^ double positive transition stage [19] and in regulating double positive thymocyte survival [21]. In addition to its function in T cell development, TCF-1 also regulates T cell differentiation. Although T cell activation seems normal in the absence of TCF-1 [19], *TCF-1^-/-^* T cells exhibit defects in differentiation to Th2 [25]. TCF-1 promotes Th2 through direct transcriptional activation of GATA-3, a master gene for Th2 differentiation, but likely does not act by affecting Th2 differentiation potential during thymic development, as such an effect can be observed by forced expression of dominant negative TCF-1 in wild-type (WT) mature T cells [25]. Further support for this idea comes from the observation that β-catenin, the TCF-1 co-activator, enhanced TCF-1-mediated stimulation of Th2 differentiation when expressed in WT mature T cells. Therefore, the question still remains whether TCF-1-mediated function in thymus eventually affects T cell function once the cells migrate into the periphery.

In this study, we demonstrate that TCF-1 is required to epigenetically maintain the IL-17 gene locus in a repressive state. Deletion of TCF-1 opens the IL-17 locus during T cell development, resulting in a high potential for differentiation of T cells into Th17 cells. Enhanced Th17 formation by deletion of TCF-1 was also demonstrated by increased susceptibility of *TCF-1^-/-^* mice to EAE induction. Furthermore, susceptibility to EAE was also observed when *TCF-1^-/-^* T cells were adoptively transferred into *Rag1^-/-^* mice, indicating that TCF-1 is intrinsically required to repress IL-17 production. However, forced expression of WT TCF-1 or dominant negative TCF in mature T cells could not interfere with IL-17 expression, suggesting that TCF-1 modulates the IL-17 locus only during the development stage. Once T cells are mature, TCF-1 can no longer affect IL-17 expression. This idea is further enhanced by our finding that forced expression of TCF-1 in *TCF-1^-/-^* mature T cells did not change the phenotype of enhanced IL-17 production. Therefore, our results demonstrate that TCF-1 regulates the developmental program to repress the IL-17 locus, resulting in modulation of the potential for Th17 formation of mature T cells.

## Materials and Methods

### Ethics Statement

All mice were housed under specific pathogen-free conditions and experiments were performed in accordance with a protocol approved by the Institutional Animal Care and Use Committee at the Beckman Research Institute of City of Hope (IACUC#07023).

### Mice

*TCF-1^-/-^* mice [26] and β-catenin transgenic (β-cat^Tg^) mice [23] were described previously. *Rag1^−/−^* mice were initially obtained from Dr. Defu Zeng (Beckman Research Institute of City of Hope). All mice were housed under specific pathogen-free conditions and experiments were performed in accordance with a protocol approved by the Institutional Animal Care and Use Committee at the Beckman Research Institute of City of Hope. The approval ID is IACUC#07023.

### T-cell isolation and differentiation

Naïve (CD4^+^CD25^−^CD44^low^CD62L^hi^) T cells were isolated from lymph nodes and spleens of mice by cell sorting using a FACSAiraII cell sorter (BD Bioscience, San Jose, CA). *In vitro* differentiation of naïve T cells into ThN or Th17 cells was done as described [27]. In brief, 2×10^5^ cells were stimulated with 0.25 µg/mL anti-CD3 (145-2C11, eBioscience, San Diego, CA) and 1 µg/mL anti-CD28 (37.51, eBioscience) in goat-anti-hamster IgG (0.2 mg/mL, MP Biomedicals, Santa Ana, CA) pre-coated 24-well plates. For ThN cells, no further supplements were added; for Th17 cells, 10 µg/mL anti-IFN-γ (XMG1.2, BioXcell, West Lebanon, NH), 10 µg/mL anti-IL-4 (11B11, BioXcell), 20 ng/mL mouse IL-6 (Peprotech, Rocky Hill, NJ) and 0.5 ng/mL human TGF-β1 (Peprotech) were added. T cells were cultured for 3 d in RPMI1640 (Cellgro, Mediatech, Manassas, VA, USA) containing 2 mM L-glutamine, 50 µM β-mercaptoethanol, 100 U/mL penicillin, 100 µg/mL streptomycin and 10% FBS.

### Intracellular cytokine measurements and flow cytometry

For cytokine analysis, cultured ThN or Th17 cells, or freshly isolated thymocytes and peripheral lymphocytes, were stimulated with 10 nM PMA and 1 µM ionomycin (Sigma-Aldrich, St. Louis, MO) for 4 h in the presence of 5 µM Brefeldin A. Antibodies were purchased from eBioscience, unless otherwise indicated. Following Fc block with anti-CD16/32 (clone 93), cells were incubated with anti-mouse CD4 (clone L3T4) and anti-mouse TCR-β (clone H57-597, BD Bioscience) and fixed/permeabilized either with the BD Cytofix/Cytoperm kit (BD Bioscience) or Foxp3 Staining Buffer (eBioscience). Cells were intracellularly stained with the following antibodies: anti-mouse IL-17A (clone eBioTC11-18H10.1), anti-mouse RORγt (clone AFKJS-9) and anti-mouse Foxp3 (clone FJK-16S), and analyzed using a BDCantoII cytometer (BD Biosciences) and FlowJo software (Treestar, Ashland, OR).

### Isolation of lamina propria lymphocytes

Method was adopted from Weigmann B (Nature protocol 2007). In brief, after carefully removal of Peyer's patches, fat tissue, and feces, small intestines were cut longitudinally first, then cut into 1 cm pieces. After two rounds of incubation at 37°C for 20 min in PBS with 5 mM EDTA and 1 mM DTT, gut tissues were washed with cold PBS and cut into 1 mm^2^ pieces. Tissues were incubated with 0.5 mg/ml collagenase D (Sigma-Aldrich), 0.5 mg/ml DNaseI (Sigma-Aldrich) and 3 mg/ml Dispase II (Roche) in PBS with 4% FBS for 20 min at 37°C with agitation. After incubation, tissues were forced to pass 40 µM filter and precipitated to pellet. Cells were further stained with anti-CD4-FITC and anti-TCR-β-PE (eBioscience) and sorted as CD4+TCR-β+ cells by BDFACSAria II.

### Retroviral Packaging and Transduction

MSCV-IRES-EGFP based retroviral bicistronic expression vectors encoding TCF-1, dominate negative TCF-1 (dnTCF-1), β-catenin or constitutively active β-catenin were transfected into phoenix cells using lipofectamine 2000 (Invitrogen). After 48 hours, viral supernatants were collected and stored at −80°C until use. For transduction, naïve T cells were first activated with anti-CD3 (1 ug/ml) and anti-CD28 (1 ug/ml) antibodies for 24 hours, then spin infected with viral supernatants (2500 rpm, 30°C for 2 hours) in the presence of 8 ug/ml of polybrene (Sigma-Aldrich). After spin infection, cytokines were added in culture media for T cell differentiation.

### EAE

EAE was induced as described [27]. Briefly, mice were immunized with 200 µL myelin oligodendrocyte glycoprotein 35–55 (MOG_35–55_) peptide emulsion (Hooke Laboratories, Lawrence, MA). On days 0 and 2 after immunization, mice were injected with 200 ng pertussis toxin (List Biological Laboratories, Campbell, CA). The severity of EAE was monitored and evaluated on a scale from 0 to 5 according to Hooke Laboratories' guideline; in brief: 0 =  no disease; 1 =  paralyzed tail; 2 =  hind limb weakness; 3 =  hind limb paralysis; 4 =  hind and fore limb paralysis; 5 =  moribund and death. When a mouse was euthanized because of severe paralysis, a score of 5 was entered for that mouse for the rest of the experiment.

### CD4 T cell Transfer

CD4 T cells were isolated with purity greater than 95% from spleens and lymph nodes of WT and *TCF-1^-/-^* mice, by negative selection using a CD4 selection kit and MACS Separation Columns (Miltenyi Biotec, Auburn, CA). WT CD4 cells (1×10^6^ or 3×10^6^ per mouse) or *TCF-1^-/-^* CD4 cells (1×1 0^6^ per mouse) were injected intravenously into unirradiated *Rag1^-/-^* mice. EAE was induced 24 h after cell transfer.

### *Ex vivo* stimulation of MOG_35–55_-specific T cells

Mice were immunized with 200 µl MOG_35–55_ emulsion without injection of pertussis toxin. Twelve days later, cells were isolated from lymph nodes and spleen and stimulated with 40 µg/mL MOG_35–55_ in the presence of 10 ng/mL IL-23 (eBioscience) for 3 d. Viable lymphocytes were isolated by Ficoll-Paque centrifugation and analyzed for IL-17A production by flow cytometry.

### STAT3 phosphorylation

Freshly isolated naïve T cells were incubated (30 min, 37°C, 5% CO_2_) with or without 20 ng/mL IL-6. Cells were fixed in 4% paraformaldehyde, permeabilized with 90% methanol (−20°C) and washed with PBS containing 1% BSA. Following Fc block with anti-CD16/32, cells were incubated with anti-mouse CD4 (L3T4, eBioscience) and anti-mouse STAT3 (pY705, clone 4/P-STAT3, BD Biosciences).

### Real-time PCR

Quantitative RT-PCR was performed as described [28]. Briefly, Total RNA was extracted from T cells or thymocytes with the RNeasy Mini Kit (Qiagen, Germantown, MD). cDNA was synthesized from total RNA using oligo(dT) primers and the Superscript III First-Strand kit (Invitrogen, Carlsbad, CA). Gene-specific cDNAs were amplified using the Ssofast EvaGreen Supermix (Bio-Rad, Hercules, CA) and a CFX thermocycler (Bio-Rad). Cycling conditions were 95°C for 30 s followed by 40 cycles of 95°C for 2 s, 60°C for 5 s. EvaGreen signals were captured at the end of each annealing and extension step (60°C). Threshold cycles for each transcript (C_T_) were normalized to GAPD (ΔC_T_). Gene expression is shown as 0.5^ΔCT^ × 10^6^. Real-time PCR reactions were performed in triplicate. Primer sequences are as following: IL-17A: 5′-CTCCAGAAGGCCCTCAGACTAC-3′, 5′-AGCTTTCCCTCCGCATTGACACAG-3′; IL-17F: 5′-GAGGATAACACTGTGAGAGTTGAC-3′, 5′-GAGTTCATGGTGCTGTCTTCC-3′; IL-23R: 5′-TCAGTGCTACAATCTTCAGAGG-3′, 5′-GCCAAGAAGACCATTCCCGA-3′; RORγt: 5′-CCGCTGAGAGGGCTTCAC-3′, 5′-TGCAGGAGTAGGCCACATTACA-3′; RORα: 5′-CAATGCCACCTACTCCTGTCC-3′, 5′-GCCAGGCATTTCTGCAGC-3′; Ahr: 5′-AGCAGCTGTGTCAGATGGTG-3′, 5′-CTGAGCAGTCCCCTGTAAGC-3′; Runx1: 5′-TACCTGGGATCCATCACCTC-3′, 5′-GACGGCAGAGTAGGGAACTG-3′; Est-1: 5′-CGGGTCCCCTCCTATGACAG-3′, 5′-GAATGACAGGCTTGTCCTTGTT-3′; Socs3: 5′-AGCTCCAAAAGCGAGTACCA-3′, 5′-TGACGCTCAACGTGAAGAAG-3′; IRF4: 5′-GCAATGGGAAACTCCGACAGT-3′, 5′-CAGCGTCCTCCTCACGATTGT-3′; Batf: 5′-CCAGAAGAGCCGACAGAGAC-3′, 5′-GAGCTGCGTTCTGTTTCTCC-3′.

### Quantitative RT-PCR-based Chromatin Immunoprecipitation (ChIP) Assay

Quantitative RT-PCR-based ChIP assay was performed with the ChampionChIP kit (SABiosciences, Qiagen). Approximately 1×10^7^ cells were used per ChIP. DNA recovered from an aliquot of sheared chromatin was used as the input sample. The remaining chromatin was precleared with protein A-agarose beads and then incubated (overnight, 4°C) with anti-acetyl-histone H3 (Cat. # 06-599, Upstate, Millipore, Temecula, CA) or anti-trimethyl-histone H3-Lys4 antibodies (Cat. # 07-473, Upstate, Millipore). Input DNA and DNA recovered after immunoprecipitation were analyzed by RT-PCR as described above, with primer pairs for IL-17A promoter: 5′-GCAGCAGCTTCAGATATGTCC-3′, 5′-TGAGGTCAGCACAGAACCAC-3′; for IL-17 conserved noncoding sequence 5 (CNS-5): 5′-AGGCCCACAATGTAGGTCAG-3′, 5′-CAGGCTGGGAAGTCTCTCTG-3′ [29]; and β-actin promoter: 5′-CAGCCAACTTTACGCCTAGC-3′, 5′-TTTGGACAAAGACCCAGAGG-3′. Results for the IL-17 locus were first normalized to the β-actin promoter (ΔC_T_), and further normalized to the level of the IL-17 locus in input fraction. Data are presented as 0.5^(ΔCT-ΔCTinput)^×10^6^.

### Statistical analysis

Statistical analysis was performed using the unpaired, two-tailed Student's *t*-test.

## Results

### Deletion of TCF-1 leads to up-regulated IL-17 gene expression

**Figure 1:**
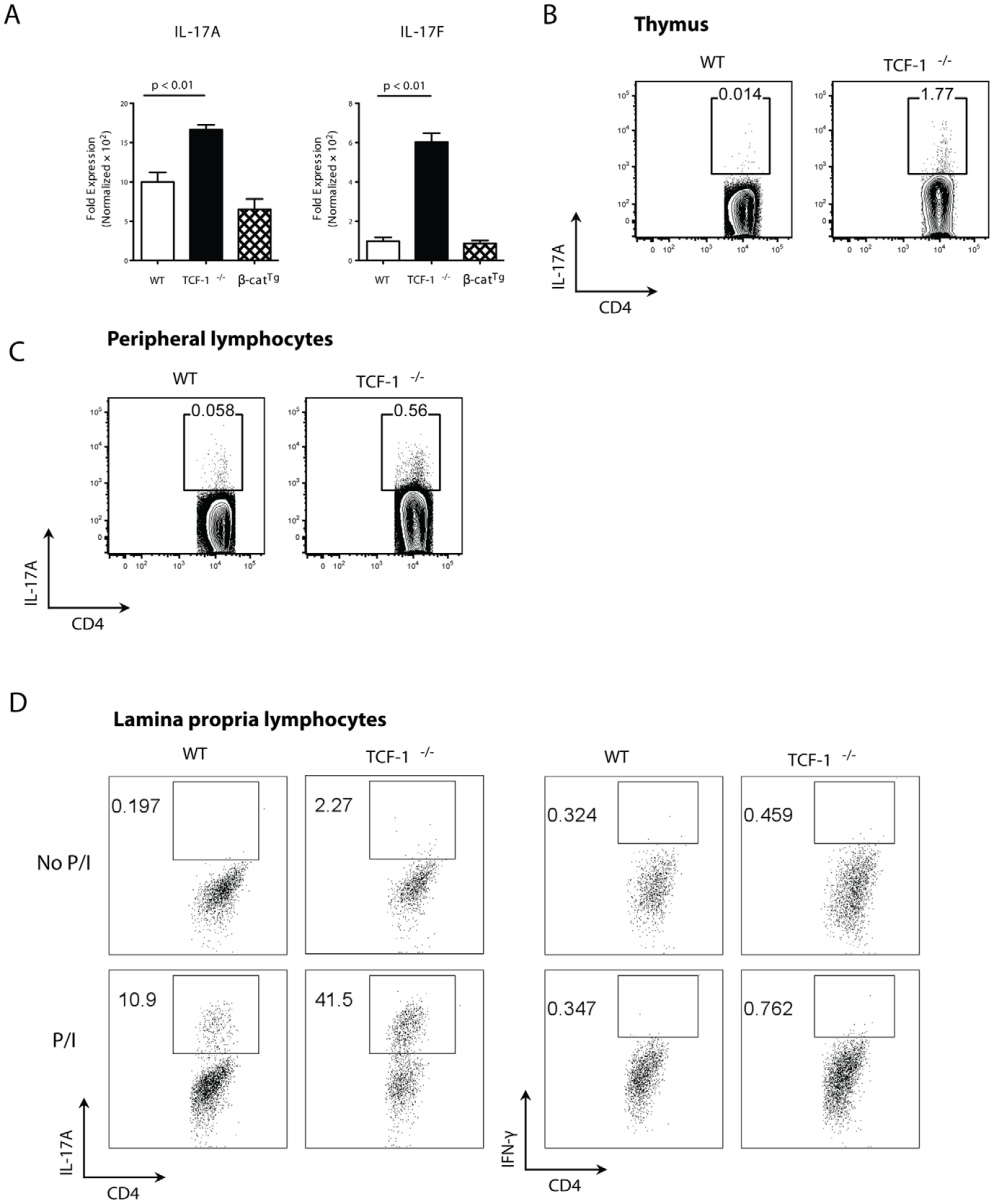
TCF-1 is required to repress the IL-17 gene during T cell development. (A) Increased expression of IL-17 mRNA in *TCF-1^-/-^* but not *β-cat^Tg^* thymocytes. Levels of IL-17A and IL-17F mRNAs from WT, *TCF-1^-/-^* or *β-cat^Tg^* thymocytes, as determined by qRT-PCR; n = 4; error bars indicate ±SEM. (B-C) Increase in natural Th17 cells in *TCF-1^-/-^* mice. T cells from thymus (B) or peripheral lymphocytes (C) of WT or TCF-1^-/-^ mice were stimulated with PMA and ionomycin for 4 h immediately after isolation, and the percentage of natural Th17 gated on CD4^+^TCR-β^+^ cells was detected by intracellular staining for IL-17. Shown contour plots are representative of three independent experiments. (D) Increased IL-17 but not IFNγ producing T cells in lamina propria lymphocytes. Lamina propria lymphocytes from WT and *TCF-1^-/-^* mice were either not treated (top panels) or stimulated with PMA/Ionomycin (bottom panels). IL-17 and IFNγ producing cells were detected by flow cytometry.

Previously, we and others demonstrated that the β-catenin/TCF-1 pathway regulates thymocyte survival [21], [23]. To study the mechanisms responsible for TCF-1-regulated thymocyte function, we performed microarray analysis of thymocytes obtained from WT, *TCF-1^-/-^* and transgenic mice that expressed a stabilized β-catenin (β-cat^Tg^). Thus, we could compare the gene expression profiles among cells in which the TCF-1 pathway was inhibited by deletion of TCF-1 and cells in which the TCF-1 pathway was activated by β-catenin. Two IL-17 genes, IL-17A and IL-17F were up-regulated in the absence of TCF-1 (data not shown), which was confirmed by quantitative real time-PCR (qRT-PCR) analysis (Fig. 1A). However, expression of neither gene was obviously changed in *β-cat^Tg^* mice compared to WT mice. Because IL-17A and IL-17F are critical cytokines produced by Th17 cells and have been linked to numerous autoimmune diseases [3], [14], we further investigated the role of TCF-1 in Th17 differentiation. Marks *et al.* described a population of cells that naturally produced IL-17 without differentiation, called natural Th17 cells (nTh17), which are present in both thymus and periphery [30]. We therefore analyzed this population of cells (Fig. 1B-1C). Because nTh17 cells are not induced, they are a relatively small population of cells; however, consistent with qRT-PCR analysis, nTh17 cells were significantly enriched (∼10-fold) in *TCF-1^-/-^* thymus (Fig. 1B). Similarly, significantly more nTh17 cells were also detected in *TCF-1^-/-^* spleen compared to that of WT mice (Fig. 1C). In addition, we examined lamina propria lymphocytes for IL-17 and IFNγ producing T cells (Fig. 1D). Prior to stimulation, *TCF-1^-/-^* mice already had more Th17 cells (2.27%) than the WT mice (0.197) (left two panels). After PMA/Ionomycin stimulation which amplified cytokine signals (second two panels), 41.5% of all the *TCF-1^-/-^* T cells were IL-17 positive, whereas only 10.9% of WT T cells were producing IL-17. In contrast, there were no obvious differences between WT and *TCF-1^-/-^* T cells in IFNγ producing cells either prior (third two panels) or after stimulation (right two panels). These results suggest that TCF-1 is required to suppress IL-17 expression.

### *TCF-1^-/-^* T cells have high potential to form Th17 cells

**Figure 2:**
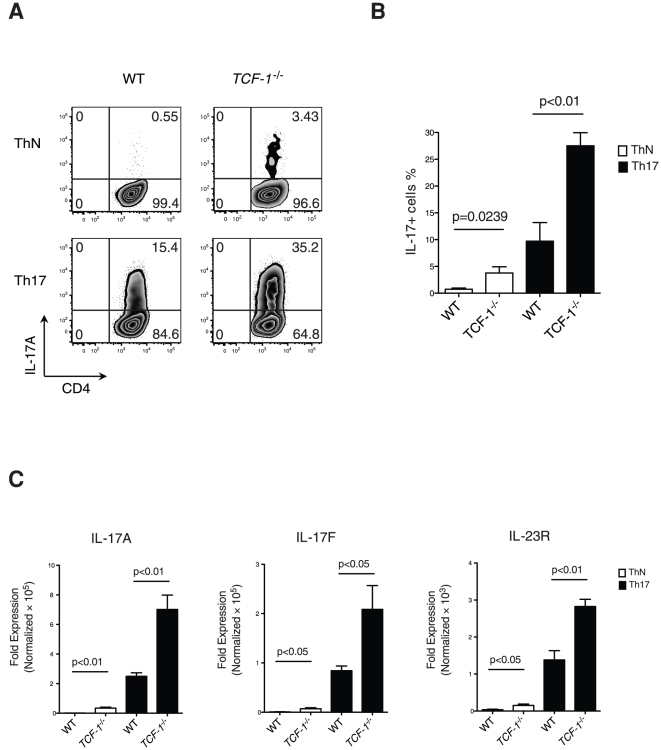
*TCF-1^-/-^* T cells have a high potential to form Th17 cells. (A) Isolated naïve CD4^+^ T cells from WT control or *TCF-1^-/-^* mice were differentiated in neutral conditions (without cytokines) or Th17 conditions (TGFβ + IL-6) for three days. Intracellular IL-17A was then stained and analyzed by flow cytometry. (B) Percentage of IL-17A producing cells as averaged (mean±SEM) from six independent experiments performed as described in A. (C) mRNA levels of IL-17A, IL-17F and IL-23R in ThN and Th17 cells described in A were determined by quantitative RT-PCR; mean±SEM from four independent experiments performed in triplicate.

To study the role of TCF-1 in Th17 differentiation, the abilities of WT and *TCF-1^-/-^* T cells to differentiate into Th17 cells were compared. T cells were stimulated in the presence or absence of TGFβ and IL-6, critical cytokines required for Th17 formation (Fig. 2A-2B). Remarkably, even in the absence of TGFβ and IL-6 (Th neutral condition, ThN), a significant percentage of *TCF-1^-/-^* T cells became Th17 cells compared to the WT T cells (Fig. 2A, top panel, and 2B). In the presence of TGFβ and IL-6, *TCF-1^-/-^* T cells produced about 100% more Th17 cells than did WT T cells (Fig. 2A, bottom panel, and 2B). qRT-PCR analysis confirmed an increase in IL-17A and IL-17F mRNAs under both the ThN and Th17 differentiation conditions in *TCF-1^-/-^* cells (Fig. 2C). In addition, we examined IL-23 receptor (IL-23R) expression, which is up-regulated in Th17 cells [31]. The increased number of Th17 cells in the absence of TCF-1 was consistently associated with increased expression of IL-23R. Taken together, our results demonstrate the potential for Th17 formation is greatly enhanced in the absence of TCF-1.

### *TCF-1^-/-^* mice are susceptible to EAE

**Figure 3:**
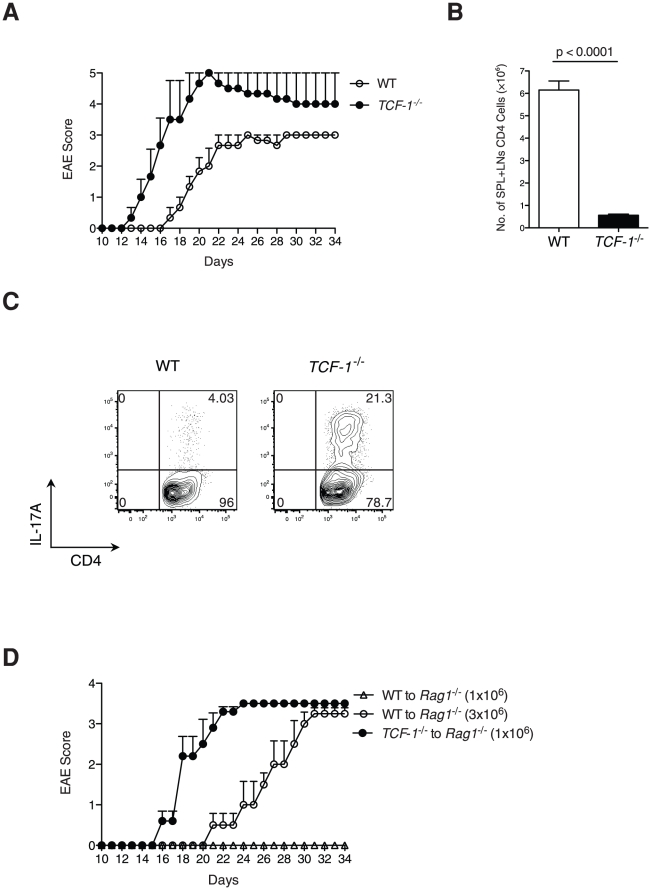
*TCF-1^-/-^* mice are susceptible to EAE. (A) EAE was induced by direct immunization of WT control and *TCF-1^-/-^* mice with MOG_35–55_, and the EAE score was determined as described in Materials and Methods; n = 3 for each group; error bars indicate ±SD. (B) Reduced number of T cells in *TCF-1^-/-^* mice. Numbers of peripheral CD4 cells in WT control or *TCF-1^-/-^* mice; n = 4; error bars indicate ±SEM. (C) Increased Th17 cells in *TCF-1^-/-^* mice. Peripheral lymphocytes were restimulated with MOG_35–55_ and IL-23 after isolation from MOG_35–55_-immunized WT control and *TCF-1^-/-^* mice. IL-17A was measured by intracellular staining. Shown contour plots are representative of three independent experiments. (D) *Rag1^-/-^* mice adoptively transferred with *TCF-1^-/-^* T cells are susceptive to EAE induction. 1×10^6^ or 3×10^6^ WT CD4 cells, or 1×10^6^ *TCF-1^-/-^* CD4 cells were transferred into *Rag1^-/-^* mice. One day later, mice were immunized with MOG_35–55_ to induce EAE; n = 6 for each group; error bars indicate ±SEM.

To determine whether TCF-1 regulates Th17 function *in vivo*, we used an EAE mouse model, Th17-dependent autoimmunity [5]. EAE was induced in both WT and *TCF-1^-/-^* mice by immunization with the MOG_35–55_ peptide. WT mice developed initial symptoms of EAE (limp tail) about 16 day after immunization, whereas *TCF-1^-/-^* mice developed EAE symptoms much earlier at day 12 (Fig. 3A). WT C57BL/6 mice developed mild EAE, with a clinical score of approximately 3. In contrast, *TCF-1^-/-^* mice developed more severe EAE, with clinical score up to 5. This result is even more dramatic considering that *TCF-1^-/-^* mice had significantly fewer peripheral T cells as compared to WT mice (Fig. 3B). In addition, we consistently detected a significantly higher percentage of IL-17A-producing CD4 T cells in *TCF-1^-/-^* mice than in WT mice (Fig. 3C), suggesting the enhanced Th17 response contributed to the more severe EAE observed in *TCF-1^-/-^* mice. Because all tissues of the *TCF-1^-/-^* mice had the gene knockout, we performed adoptive transfer experiments to specifically determine the function of TCF-1 in T cells during the development of EAE (Fig. 3D). EAE was induced in *Rag1^-/-^* mice that were adoptively transferred with 1×10^6^ or 3×10^6^ WT CD4 T cells or 1×10^6^ *TCF-1^-/-^* CD4 T cells. Adoptive transfer of 1×10^6^ WT T cells was not sufficient to induce EAE, but transfer of 3×10^6^ WT T cells was able to induce EAE. In contrast, transfer of 1×10^6^ *TCF-1^-/-^* T cells induced EAE more severely than that of 3×10^6^ WT T cells. These results demonstrated that deletion of TCF-1 led to increased susceptibility to EAE, likely due to enhanced Th17 responses. Thus, TCF-1 is required *in vivo* to repress Th17-dependent immune responses.

### Expression of factors known to regulate Th17 differentiation are not affected by lack of TCF-1

**Figure 4:**
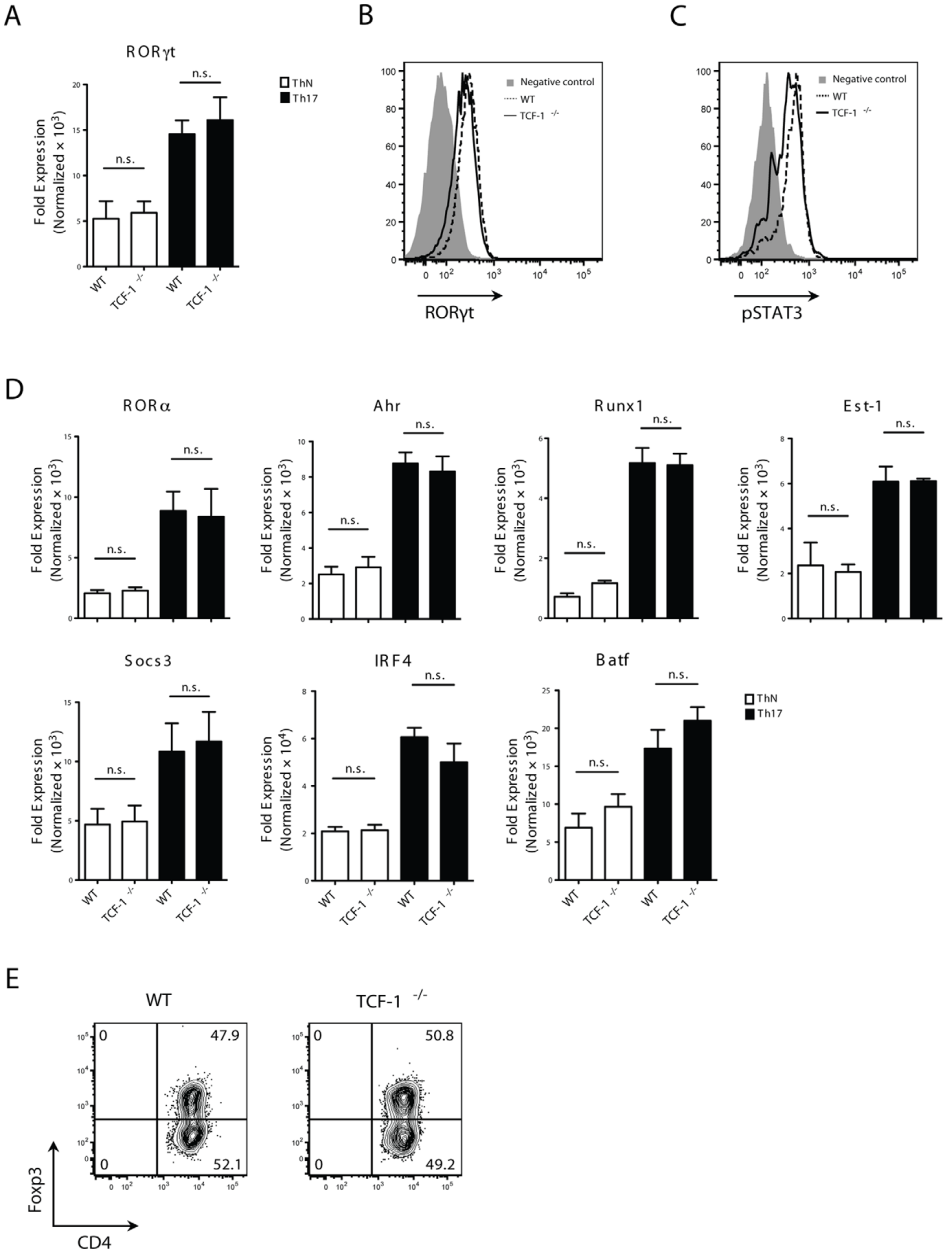
*TCF-1^-/-^* cells do not have significant changes in expression of factors known to regulate Th17. (A) qRT-PCR analysis of mRNA levels of RORγt in ThN and Th17 conditions; mean±SEM from four independent experiments performed in triplicate. (B) *TCF-1^-/-^* cells normally up-regulates RORγt. Expression of RORγt in WT or *TCF-1^-/-^* Th17 cells as determined by intracellular staining. *RORγt^-/-^* T cells cultured under Th17 condition were used as negative controls for RORγt staining (shaded area). (C) *TCF-1^-/-^* cells normally activate Stat3. Naïve WT or *TCF-1^-/-^* CD4 cells were incubated with or without IL-6 for 30 min, fixed and stained with anti-STAT3 (pY705) antibody and pSTAT3 levels were determined by flow cytometry; n = 3. (D) *TCF-1^-/-^* cells normally up-regulates factors known to regulate Th17 differentiation. qRT-PCR analysis of mRNA levels of RORα, Ahr, Runx1, Est-1, Socs3, IRF4 and Batf in ThN and Th17 cells; mean±SEM from four independent experiments performed in triplicate. (E) Normal differentiation of *TCF-1^-/-^* cells to iTreg. Naïve WT or TCF-1^-/-^ CD4 cells were cultured with anti-CD3/CD28 and 5 ng/ml TGF-β1 to induce Foxp3 expression. Foxp3 expression was determined by flow cytometry after three days of culture; n = 3.

To determine the molecular mechanisms responsible for TCF-1-regulated Th17 differentiation, we assessed factors known to regulate Th17 differentiation. qRT-PCR analysis of RORγt, a master regulator for Th17 [17], did not detect an obvious difference in RORγt expression between WT and *TCF-1^-/-^* T cells, either under ThN or Th17 differentiation conditions (Fig. 4A). Flow cytometric analysis of intracellularly stained RORγt confirmed this result (Fig. 4B). Because Stat3 is another important factor involved in regulating Th17 differentiation, we used an anti-phosphorylated Stat3 antibody to examine activation of Stat3. No difference in expression of phosphorylated Stat3 was detected between WT and *TCF-1^-/-^* T cells (Fig. 4C), suggesting normal activation of Stat3 in the absence of TCF-1. qRT-PCR also revealed no difference in expression of RORα [18], Ahr [32], Runx1 [33], Ets-1 [29], [34], [35], Socs3 [36], IRF4 [37], or Batf [38], factors that have been reported to positively or negatively regulate Th17 differentiation (Fig. 4D). Lastly, TGFβ-induced Foxp3 has been shown to inhibit RORγt, a mechanism believed to control Treg-Th17 balance. We therefore examined TGFβ-induced Treg, and lack of TCF-1 did not affect Treg differentiation (Fig. 4E). Thus, TCF-1 seems to regulate Th17 differentiation without affecting the above factors known to influence Th17 formation.

### TCF-1 fails to affect Th17 differentiation in mature T cells

**Figure 5:**
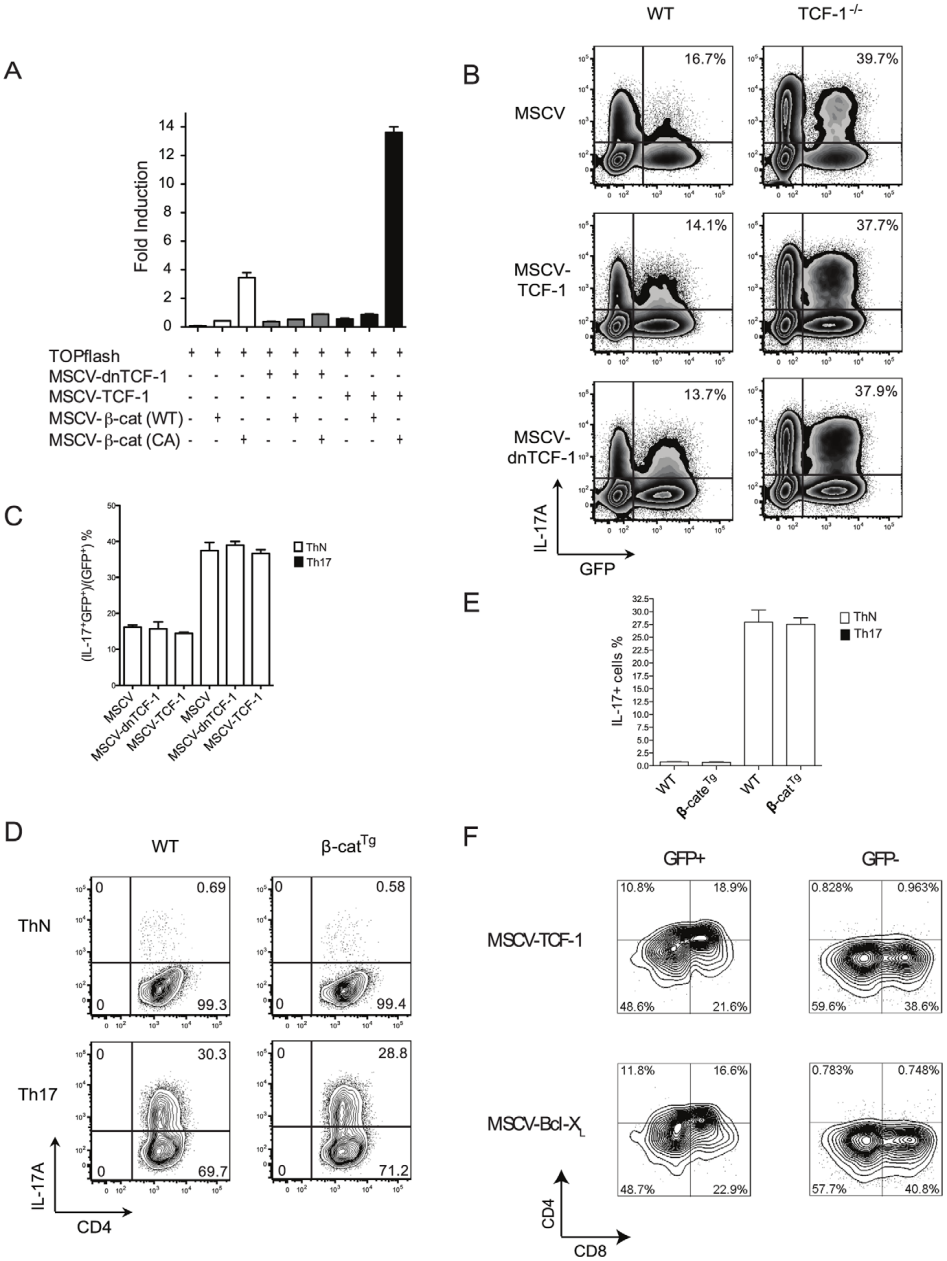
TCF-1 does not affect IL-17 expression in mature T cells. (A) TCF-1, dnTCF-1, β-catenin, or active β-catenin expressed from MSCV plasmids functioned normally in the regulation of a TCF-1 reporter. HEK293 cells were co-transfected with TOPflash reporter and MSCV-TCF-1, dnTCF, β-catenin, or active β-catenin vectors, as indicated. Luciferase activity was measured 48 h after transfection and normalized to internal control Renilla luciferase; n = 2. (B) Expression of TCF-1 or dnTCF does not affect Th17 differentiation in either peripheral WT or *TCF-1^-/-^* CD4 cells. Naïve WT or TCF-1^-/-^ CD4 cells were infected with empty MSCV, MSCV-dnTCF-1 or MSCV-TCF-1, then differentiated under Th17 conditions. Production of IL-17A was determined by intracellular staining. Percentage shown at the right upper corner was calculated as (IL-17+GFP+)/(GFP+)× 100%. (C) Percentage of IL-17^+^GFP^+^ cells as shown in (B); n = 2 for each group. (D) Active β-catenin does not affect Th17 differentiation. Isolated naïve CD4 T cells from WT control or *β-cat^Tg^* mice were cultured under ThN or Th17 condition for three days. Representative flow cytometry plots of intracellular IL-17A staining are shown. (E) Percentage of IL-17A-producing cells as shown in (D); n = 2 for each group. Error bars indicate ±SEM. (F) Retrovirally expressed TCF-1 rescued defective *TCF-1^-/-^* thymocyte development. CD4-CD8- thymocytes sorted from WT and *TCF-1^-/-^* thymocytes were co-cultured with stroma cells OP9-DL1. After 24 hrs, retrovirus expressing TCF-1 or Bcl-x_L_ transduced cells, and allowed to differentiate for three more days. T cell development of GFP+ and GFP- cells was then analyzed using CD4 and CD8 surface markers by flow cytometry.

We next investigated whether TCF-1 can rescue Th17 differentiation of *TCF-1^-/-^* T cells. A murine stem cell virus (MSCV)-based retrovirus system was used to express TCF-1 and β-catenin. As a control, a dominant negative TCF (dnTCF) that can interfere with WT TCF-1 activity was also used. We first used a TCF reporter (TOPflash) system to confirm that the TCF-1, dnTCF and β-catenin plasmids functioned normally (Fig. 5A). β-catenin stimulated the TCF-1 reporter, and stabilized active β-catenin stimulated TCF-1 to a higher level. In the presence of dnTCF, active β-catenin failed to stimulate the TCF-1 reporter due to the expected inhibition of endogenous TCF-1. The TCF-1 reporter was most stimulated in the presence of WT TCF-1 and active β-catenin. These results demonstrated that the retrovirus vector-expressed TCF-1 and dnTCF functioned as expected.

Retroviruses that expressed TCF-1 or dnTCF were then used to transduce WT or *TCF-1^-/-^* T cells grown under Th17 differentiation conditions (Fig. 5B–5C). Because MSCV contains a gene for GFP expression, cells were divided into GFP-negative (no virus infection) and GFP-positive (virus infected, and therefore expressed protein of interest) populations. First, empty virus (MSCV) was used for transduction (Fig. 5B, top panel). Consistent with previous results (Fig. 2A), significantly more Th17 cells were detected in both the GFP^-^ and GFP^+^ populations (percentage of Th17 cells among GFP^+^ cells is indicated) in *TCF-1^-/-^* T cells compared to WT cells (Fig. 5B), suggesting that virus transduction did not affect Th17 differentiation potential. TCF-1 did not affect Th17 formation in WT T cells, which was likely not a result of overexpression of TCF-1, as expression of TCF-1 in *TCF-1^-/-^* T cells also failed to affect Th17 formation (Fig. 5B, middle panel). This result also indicated that expression of TCF-1 failed to rescue the Th17 differentiation defects observed in *TCF-1^-/-^* T cells. Similarly, dnTCF, which should interfere with endogenous TCF-1, also failed to affect Th17 differentiation in either WT or *TCF-1^-/-^* T cells (Fig. 2B, bottom panel). The reproducibility of above experiments described in figure 2B was indicated by figure 2C, an average from several independent studies. In addition, we tested whether β-catenin, an activator of TCF-1, affected Th17 differentiation. T cells obtained from WT or transgenic mice that expressed stabilized β-catenin (*β-cat^Tg^*) [23] were differentiated in ThN or Th17 conditions. No differences were observed in Th17 formation between WT and *β-cat^Tg^* T cells (Fig. 5D–5E). This result was confirmed using retrovirus to express β-catenin (data not shown). As a control, we determined whether retrovirally expressed TCF-1 can rescue *TCF-1^-/-^* thymocyte development (Fig. 5F). CD4-CD8- double negative thymocytes were sorted and allowed to differentiate on stroma cells OP9-DL1 [39], [40], [41]. WT T cells can differentiate into CD4+CD8+ double positive T cells, whereas *TCF-1^-/-^* failed to do so (data not shown) due to apoptosis of *TCF-1^-/-^* CD4+CD8+ cells [21], [22]. Retrovirus expression either TCF-1 or anti-apoptotic Bcl-x_L_ rescued development of CD4+CD8+ cells (Fig. 5F, left two panels, GFP+ cells). Whereas, GFP- cells, which were not transduced and thus did not express TCF-1 or Bcl-x_L_ failed to develop into CD4+CD8+ cells. TCF-1, that rescued defective *TCF-1^-/-^* thymocyte development, failed to correct abnormal Th17 differentiation in *TCF-1^-/-^* mature T cells. Taken together, we conclude that TCF-1 is unlikely to affect Th17 differentiation potential at the mature T cell stage.

### IL-17 gene locus is in an “open” state in the absence of TCF-1

**Figure 6:**
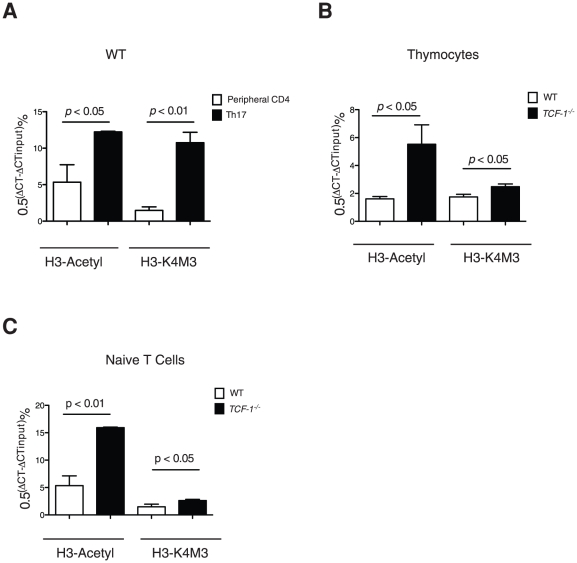
The IL-17 locus in *TCF-1^-/-^* thymocytes and peripheral T cells is in an open state. (A) IL-17 locus is opened up upon differentiation to Th17 cells. H3-Acetyl and H3-K4M, both chromatin modifications indicating active gene locus, were measured in WT naïve T cells or differentiated Th17 cells; (B-C) IL-17 locus is in open sate in *TCF-1^-/-^* cells. H3-Acetyl and H3-K4M were compared between WT or *TCF-1^-/-^* thymocytes (B) or peripheral T cells. DNA was precipitated with anti-acetyl-histone H3 (H3-Acetyl) or trimethyl-histone H3 (H3-K4M3) antibodies. qRT- PCR-based ChIP analysis was used to measure acetyl-histone H3 or trimethyl-histone within the IL-17 locus; n = 4 for each group; error bars indicate ±SEM.

Since TCF-1 does not regulate IL-17 expression in mature T cells (Fig. 5), and IL-17 starts to up-regulate in *TCF-1^-/-^* thymocytes (Fig.1), it suggests a possibility that TCF-1 mediates the repression of IL-17 gene locus in thymus instead of in periphery. We thus examined histone H3 acetylation (H3-Acetyl) and methylation at Lys-4 (H3-K4M3), both chromatin modifications contributing to open up the gene locus for active transcription [42]. Previous study has demonstrated significantly increased H3-Acetyl and H3-K4M3 during Th17 differentiation, indicating opening IL-17 gene locus for expression [29]. Indeed, our results confirmed significant higher H3-Acetyl and H3-K4M3 in differentiated Th17 cells than that of the naïve T cells (Fig. 6A). We next compared both modifications between WT and *TCF-1^-/-^* mice. Higher H3-Acetyl and H3-K4M3 were observed in *TCF-1^-/-^* thymocytes (Fig. 6B) and peripheral T cells (Fig. 6C). These results suggest that lack of TCF-1 leads to opening up IL-17 locus in thymus due to chromatin modifications. Such open state of IL-17 locus is maintained in mature T cells due to preserved chromatin modification patterns, which explains why peripheral *TCF-1^-/-^* T cells have high Th17 formation potential, although TCF-1 cannot interferes with IL-17 production in mature T cells. Altogether, our results demonstrate that TCF-1 mediates the repression of IL-17 locus during T cell development by chromatin modifications.

## Discussion

Naïve T cells are pluripotent and able to differentiate into various lineages of T helpers that produce distinct cytokines mediating different immune responses. Critical cytokine loci, such as IFNγ (Th1), IL-4 (Th2) and IL-17 (Th17), are repressed in naïve T cells until the engagement of antigens that initiate differentiation. Previous studies have demonstrated the IFNγ and IL-4 gene loci undergo dramatic epigenetic changes in chromatin modification, resulting in activation of their expression upon differentiation to Th1 and Th2 respectively [43]. Similarly, during Th17 differentiation, the IL-17 locus also undergoes extensive chromatin modification, leading to a significant increase in chromatin accessibility for transcription activation [29]. Peripheral mature T cells are thus capable of converting inactive cytokine gene loci to active. Such a capability requires that the cytokine gene loci be programmed into a reversible repressive state during T cell development so that they can be de-repressed and further activated once T cells migrate out of the thymus. However, little is known about the mechanisms responsible for programming cytokine gene loci during T cell development. In this study, we demonstrated that TCF-1 is a critical factor required to repress IL-17 gene locus in thymus. Compared to WT mice, mice lacking TCF-1 had increased IL-17 gene expression both in thymic and peripheral T cells, which was confirmed by a larger natural Th17 cell population in these mice (Fig. 1B-C). Furthermore, when stimulated *in vitro*, even in the absence of TCFβ and IL-6, cytokines critical for Th17 formation, *TCF-1^-/-^* T cells formed Th17 cells, suggesting an elevated potential for Th17 formation in the absence of TCF-1. Indeed, *TCF-1^-/-^* T cells formed twice as many Th17 cells than did WT cells in the presence of TGFβ and IL-6. Lastly, *TCF-1^-/-^* mice and *Rag1^-/-^* mice adoptively transferred with *TCF-1^-/-^* T cells were more susceptible to induction of EAE, a Th17-dependent autoimmune disease. Taken together, our *in vitro* and *in vivo* experiments support that TCF-1 is intrinsically required to suppress Th17 differentiation. Consistent with our results, a recent study also demonstrated negative role of TCF-1 in Th17 differentiation [44]. However, it was suggested that TCF-1 likely regulate IL-17 at mature stage. In contrast, our results support that TCF-1 modifies IL-17 gene locus during T cell development in thymus, and such modifications are imprinted in cells even after T cells mature and migrate out of thymus. Our data do not exclude other alternative explanation. Since TCF family has multiple members including TCF-3, TCF-4 as well as LEF-1, we do not exclude the possibility that T cells at mature stages use different members of TCF to regulate Th17, which could explain why TCF-1 does not affect Th17 differentiation at mature stage. However, dnTCF, which also interferes other TCF function, did not affect Th17 differentiation in mature stage, favoring that TCF-1 regulates IL-17 locus in immature stage. In addition, we would like to mention that only mice were used in this study. The mechanisms revealed in this study may not apply to human T cells, as human T cells are not as stringent in terms of lineage commitment in comparison to mouse T cells.

Although many transcription factors have been identified as either positively or negatively regulating Th17 differentiation, including RORγt [17], Stat3 [45], Runx1[33], IRF4 [37], Ahr [32], Batf [38], Foxp3 [46] and Ets-1 [29], [34], [35], TCF-1 is unique because it regulates Th17 differentiation without affecting RORγt (Fig. 4A–B), whereas other transcription factors promote or inhibit Th17 differentiation by regulating the levels of RORγt. Furthermore, deletion of TCF-1 does not alter expression of the above listed factors known to regulate Th17 differentiation (Fig. 4D). In addition, TCF-1 does not affect Th17 differentiation in mature peripheral T cells. We showed that overexpression of WT TCF-1, dnTCF or the TCF-1 coactivator β-catenin did not interfere with Th17 differentiation, which was not due to overexpression of TCF-1, as restoration of TCF-1 expression in mature *TCF-1^-/-^* T cells also failed to interfere with Th17 differentiation (Fig. 5B–C). In contrast, restoring expression of factors that affect Th17 differentiation in their corresponding knockout T cells usually restores IL-17 production [17], [35], [45]. These results exclude the possibility that TCF-1 regulates Th17 differentiation potential in mature T cells, which raises the question of when and where TCF-1 determines Th17 differentiation potential.

Many previous studies focused on how the cytokine environment determines the differentiation of mature T cells to specific lineages of T helpers. However, T cell function is also shaped during development in the thymus. The best example is the positive and negative selection process in thymus to ensure the specificity of T cell response to only foreign antigens [47]. Once T cells are mature and migrate out of thymus, the specificity of T cell response to antigens cannot be changed. Little is known about whether and how T cell development in thymus affects the potential of mature T cells to differentiate into a specific lineage of T helpers. Our study supports the idea that TCF-1 determines the Th17 differentiation potential during T cell development in the thymus, but not in the periphery after maturation of T cells, and thus indicates that the T cell development process can also shape the potential of mature T helper cell differentiation, which is critical for generating balanced immune responses and avoiding autoimmunity.

T cell development is a complicated process that depends on reprogramming chromatin structure to activate and repress a network of genes critical for specification and function of T cells. Acetylation and methylation are two critical epigenetic modifications of DNA for regulating gene expression by changing the accessibility of the chromatin [42]. Chromatin remodeling of the IL-17 locus has been detected during Th17 differentiation [29], and in naïve T cells this locus is hypoacetylated (H3-Acetyl) and hypomethylated at Lys-4 of histone 3 (H3-K4M3), indicating a closed state. During Th17 differentiation, the IL-17 locus is slowly transformed into an open state indicated by hyper-H3-Acetyl and hyper-H3-K4M3. Our results demonstrate that the IL-17 locus in *TCF-1^-/-^* thymocytes is more acetylated (H3-Acetyl) and slightly higher methylated (H3-K4M3) than in WT cells, suggesting an abnormal open state of the IL-17 locus, which would explain the increased levels of IL-17 mRNA and natural Th17 observed in *TCF-1^-/-^* thymocytes (Fig. 1). Thus, our results suggest there is a TCF-1-mediated active process to repress the IL-17 locus by epigenetic modifications during T cell development in thymus. The presence of increased IL-17 mRNA levels and natural Th17 in both thymic and peripheral T cells of *TCF-1^-/-^* mice further suggests that TCF-1-mediated epigenetic modifications of IL-17 locus, once finished in the thymus, can be preserved even after T cells migrate out of the thymus to the periphery. This explains our observation that peripheral *TCF-1^-/-^* T cells have high potential to form Th17 (Fig. 2), although TCF-1 could not affect Th17 differentiation in mature T cells (Fig. 5). TCF-1-mediated epigenetic modification of IL-17 locus is likely a key mechanism for preventing T cells from generating overwhelming inflammatory IL-17, which has been associated with numerous autoimmune diseases [48]. This notion is supported by the fact that *TCF-1^-/-^* mice are extremely susceptible to EAE (Fig. 3).

Much remains unknown concerning detailed molecular mechanisms responsible for TCF-1-regulated IL-17 expression. β-catenin has been reported to activate target gene expression together with TCF-1 [26], [49]. However, our results do not support the idea that β-catenin as an activator of TCF-1 is involved in this process, as a stabilized β-catenin transgene that is expressed in the thymus did not affect IL-17 differentiation (Fig. 4). TCF-1 inhibits target gene expression when it associates with the co-repressor GRG in mouse (Groucho in Drosophila) [50]. However, it has not yet been determined whether GRG is required for TCF-1 to inhibit the IL-17 locus. It is possible that TCF-1 does not directly inhibit IL-17 gene but via another factor. For example, TCF-1 may activate an IL-17 inhibitor or inhibit an IL-17 activator or both. We have demonstrated that TCF-1 is a critical factor required to inhibit IL-17 gene expression through epigenetic modifications in the thymus, which is a critical mechanism for generating appropriate Th17 responses. Disruption of this mechanism likely leads to overt Th17 inflammation that contributes to autoimmunity. TCF-1/β-catenin pathway plays a role in multiple types of immune cells including dendritic cells. For example, β-catenin has been shown to regulate Treg vs. inflammatory T cell balance via dendritic cells [51]. Therefore, TCF-1/β-catenin regulates multiple functions and multiple types of cells in immune system. More studies on how different immune cells integrate TCF-1/β-catenin signals to coordinate immune responses are essential to understanding the function of this important pathway in immune system.
